# The Function of the Hippocampus in Bridging Functional and Temporal Discontiguity

**DOI:** 10.1155/2020/1049721

**Published:** 2020-11-07

**Authors:** Ze Zhang, Kazuhisa Niki, Jing Luo

**Affiliations:** ^1^Beijing Key Laboratory of Learning and Cognition, School of Psychology, Capital Normal University, Beijing, China; ^2^Human Informatics Research Institute, Advanced Industrial Science and Technology, Tsukuba, Japan; ^3^Keio University Graduate School of Human Relations, Tokyo, Japan; ^4^Department of Psychology, Shaoxing University, Shaoxing, China

## Abstract

Theoretical assessment of the function of the hippocampus has suggested that given certain physiological constraints at both the neuronal and cortical level, the hippocampus is best suited to associate discontiguous items that occur in different temporal or spatial positions. Conceptually, “discontiguous” refers to events that are to be associated with one another but do not temporally or spatially overlap. However, given that humans can actively maintain information “online” by rehearsing it, even when the information is no longer being presented to the sensory system, the right way to experimentally define “discontiguity” is still a question. Does it refer to a “gap” in the presentation of information (temporal discontiguity) or to an “interruption” of the active maintenance of working memory (WM) information (functional discontiguity)? To assess this, participants were imaged by functional magnetic resonance imaging (fMRI) when making judgments on whether two words were semantically related or not. In contrast with recognition memory that can be carried out through perceptual familiarity heuristics, judgments on semantic relatedness can only be accomplished through associative processing. To assess this experimentally, two words are either (1) presented at the same time (Event AB) or (2) one after the other with an unfilled, cross-viewing delay (Event A_B) (the uninterrupted discontiguity) or (3) presented one after the other, between which participants are required to perform a calculation task (Event A#B) (the interrupted discontiguity). Results of event-related fMRI analysis revealed that relative to Event AB, Event A_B was not associated with more hippocampal activity, whereas Event A#B was. The direct contrast of Event A#B relative to Event A_B also revealed significant hippocampal and parahippocampal activity. This result implied that functional discontiguity (the interruption of online maintenance of the inputted information) could be more apt at engaging the function of the hippocampus.

## 1. Introduction

The hippocampus has been recognized as the important cortical region for episodic memory. Its key feature has been proposed to be the representation of event sequences [1–3]. Theories have pointed to the role of the hippocampus in associating events across time [4]. Correspondingly, computational models also demonstrated how “local context” neurons (i.e., hippocampal cells that represent events in relation to the preceding and following events) could link discontiguous events to create a network of related episodic memories [2]. In addition, study also found the “time cells” in the hippocampus that mediated the bridging of temporal gap in memory for discontiguous events [5–7]. Conceptually, this “discontiguity” refers to the events that are to be associated with one another but do not temporally or spatially overlap [2]. However, the working memory (WM) system, especially that of humans, can actively maintain information “online” even when the stimulus is no longer being presented to the sensory system. For example, one can hold a piece of verbal information online by rehearsing it, meaning that the information that is not perceptually available may not necessarily be “discontiguous.” This raises the question of how to experimentally define this concept of “discontiguity.” Does it simply refer to a “gap” in the presentation of information (temporal discontiguity) or an “interruption” of the active maintenance of information stored in WM (functional discontiguity)?

**Figure 1:**
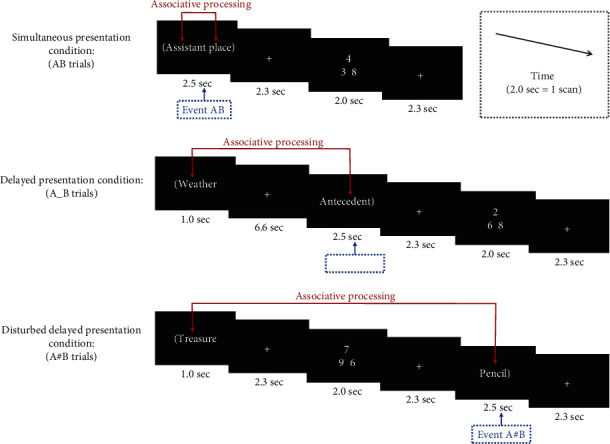
Examples of different type of trials. Notice that each pair of to-be-judged words was included in parentheses in order to avoid confusion between trials.

To assess this, we asked participants to perform a semantic relatedness judgment task during neuroimaging scanning. Participants were required to judge whether two words were semantically related or not. The two words were presented either (1) at the same time (the AB trials) or (2) one after the other separated by a cross-viewing delay (the A_B trials) or (3) one after the other, between which participants performed a calculation task (the A#B trials) (Figure 1). AB trials served as a baseline, whereas the other two conditions contained the association of the two items that did not temporally overlap with one another. In the A_B trials, the first word could be actively maintained in WM by rehearsing it during the cross-viewing delay between the display of the two words. For this reason, the first word could also be maintained “online” at the moment of associative processing elicited by the presentation of the second word. However, in the A#B trials, participants were asked to engage in a distracting calculation task between the presentation of the two words, thereby interrupting the active maintenance of the first word. In this case, the first word could be “offline” at the moment of associative processing.

So far, the role of the hippocampus in bridging temporally discontiguous events has largely been investigated and proven in animal studies [5, 6, 8]. A few neuroimaging studies in human subjects have addressed the activation of the medial temporal lobe (MTL), including the hippocampus and parahippocampus, during the association of discontiguous events [9–12]. Our previous studies investigated how pairs of temporally discontiguous events (e.g., name-face pairs or object and face pairs) were encoded into long-term memory by MTL using a subsequent memory test paradigm [11, 12]. Additionally, there were also studies that examined the involvement of the MTL in the associative processing between the continuous or discontinuous memory retrieval. For example, in Sakai et al.'s [9] study, participants memorized a sequence of five consonant letters and were asked to perform a recognition judgment after a brief delay period with or without the interruption of an arithmetic calculation. However, their results only revealed MTL activation (but only in parahippocampus) during the retrieval of letter sequence in the presence of an interruption. This was not completely consistent with animal studies that found the function of the entorhinal cortex-hippocampal network in associating events separated by time or temporal gaps [7]. One possible reason for this lack of hippocampal activation was the recognition memory task in Sakai et al.'s [9] study which could be accomplished based on the perceptual familiarity of the stimulus and did not require hippocampal engagement [13]. In contrast, our previous study did observe left hippocampal activation in an associative processing task requiring participants to judge whether two words, presented simultaneously or one after the other separated by a brief delay, were semantically related or not [10]. However, in that study, there was no distraction task inserted in the delay period between the presentation of the two words. Therefore, we still do not know whether it was the “temporal discontiguity” or the “functional discontiguity” that was responsible for activating the hippocampus. This study's design will enable us to precisely discriminate between these two factors.

It should be noted that with regard to the function of the MTL in discontiguity binding, two issues had been investigated. One relates to whether the MTL participates in the temporary or transient binding of two or more discontiguous events. The other relates to whether the MTL contributes to consolidating these (temporarily formed) discontiguity bindings into one's long-term memory. The first issue could be investigated by assessing the involvement of MTL areas during the moment of making discontiguity bindings (relative to the nondiscontiguous ones) [10, 14, 15]. In contrast, the second issue could be investigated by assessing the involvement of the MTL areas in the moment of the discontiguity binding which could be successfully remembered in the later long-term memory task (relative to the forgotten ones) [11, 12, 16]. In the present study, we only focused on the first issue and did not assign the subsequent memory test to further check if the given “discontiguity binding” was successfully maintained in long-term memory or not.

## 2. Materials and Methods

### 2.1. Participants

Twelve healthy, right-handed volunteers (6 females and 6 males, aged 20 to 31 years old) recruited from the undergraduate and graduate students of University of Tsukuba participated in the experiment as paid volunteers. They were interviewed one or two days before beginning the fMRI experiment and had provided informed consent in accordance with the Advanced Industrial Science and Technology (AIST) MRI ethics committee.

### 2.2. Cognitive Tasks

This experiment consisted of two types of cognitive tasks. One consisted of a semantic-relatedness judgment task in which participants were asked to judge whether two words were semantically related. The other consisted of a calculation task in which triples of numbers were presented, with one on the top and two on the bottom. Subjects were asked to judge which one of the two bottom numbers (the bottom left or the bottom right number) was “closest” to the top number (Figure 1). For example, the number “3” on the bottom left is closer to the top number “4” than the number “8” on the bottom right (i.e., [4–3] < [8–4]). For both tasks, participants were asked to indicate their selection by pressing the left or right key on the button box using their right index or middle finger. The left key-pressing connoted “yes/related” in the semantic relatedness judgment or “bottom left” in the calculation task; right key-pressing connoted “no/unrelated” in the semantic relatedness judgment or “bottom right” in the calculation task. To minimize judgment bias and maintain participants' arousal, in the semantic relatedness judgment, half of the word pairs in each condition contained were obviously semantically related, while half of them were not [10]. In addition, in the calculation task, there was an equal chance for the target number to appear in the bottom left as in the bottom right.

The presentation times of each stimulus are shown in Figure 1. For a trial in AB condition, the two words were presented at the same time and lasted for 2.5 sec. Participants should judge if these two words were semantically related or not during this period. Then, after a 2.3 sec cross-viewing delay, a calculation task was presented for 2 sec and followed by a 2.3 sec cross-viewing delay. For a trial in A_B condition, the first word was presented for 1 sec followed by a 6.6 sec cross-viewing delay. Then, the second word was presented for 2.5 sec and participants should make a semantic relatedness judgment during this moment. Then, after a 2.3 sec cross-viewing delay, a mental a calculation task was presented for 2 sec and followed by a 2.3 sec cross-viewing delay. For a trial in A#B condition, the first word was presented for 1 sec and followed by a 2.3 sec cross-viewing delay. Then, a mental a calculation task was presented for 2 sec and followed by a 2.3 sec cross-viewing delay. Finally, the second word was presented for 2.5 sec (participants should make a judgment) and followed by a 2.3 sec delay. In sum, in the A#B trials, the two words were presented one after the other separated by a 6.6 sec delay during which participants performed the calculation task. In the AB trials, however, the two words were presented at the same time. In the A_B trials, the two words were presented one after the other separated by a 6.6 sec unfilled, cross-viewing interval. Subjects were required to keep the first word in their mind and judge whether the first word was semantically related to the second word on seeing the second one. Calculation tasks were also assigned (but in separate ways from the session of semantic relatedness judgment task) in the AB and A_B trials to create a situation comparable to that in the A#B trials.

Ninety pairs of two-character Japanese Kanji words were used in a total of 30 trials for each of the three conditions. Word pairs were assigned counterbalanced across subjects. The 30 trials in each condition were assigned to four blocks with 7 or 8 trials in each block (in each block, the ratios of related versus unrelated word pairs were 4 : 4 in the 8-item block and 3 : 4 or 4 : 3 in the 7-item block). The whole session, therefore, consisted of a total of 12 blocks. These 12 blocks were presented in semirandom order with the constraint being that two same condition blocks (e.g., two blocks belonged to AB condition) were not to be presented continuously. These 12 task blocks were separated by three long resting (cross-viewing) blocks (block length = 25 sec) and 8 short ones (block length = 5 sec). To familiarize subjects with the procedure and pace of the task, prior to the formal experiment, they were trained with another set of similar materials performed at the same pace.

### 2.3. Data Acquisition

All imaging was performed using a 3.0-T MRI scanner (GE 3T Signa) equipped with EPI capability. Eighteen axial slices (5.3 mm thick, interleaved) were prescribed to cover the whole brain. A T2∗ weighted gradient echo EPI was used. The imaging parameters were TR = 2000 ms, TE = 30 ms, FA = 70 degrees, FOV = 20 × 20 cm (64 × 64 mesh). To reduce susceptibility noise artifacts (especially the EPI distortion) in the lower parts of the brain, including the anteromedial temporal lobes, we used a wider bandwidth (130 kHz) and moved participants' chins such as to face down. To reduce head movement, participants were asked to put on a neck brace and were requested not to talk or move during scanning. Motion correction was also performed by a standard realignment process in SPM software (Wellcome Department of Cognitive Neurology, Institute of Neurology, London, UK).

### 2.4. Data Analysis

The imaging data were analyzed using SPM software. Each participant's data was first preprocessed (timeslice adjusted, realigned, normalized, and smoothed) on an individual basis. Next, these preprocessed data were individually examined to establish first-level models (using the event-related analysis module) and then subjected to second-level, random effect models that incorporated the contrasts of all 12 participants. The aim of this study was to investigate transient brain activation in the moment of associative processing or semantic-relatedness judgment tasks. In the AB trials, the associative processing occurred when the two words were simultaneously presented, whereas in the A_B and A#B trials, the associative processing occurred when the second word was presented. Therefore, the association of discontiguous events was time-locked to the presentation of the second word in the A_B or the A#B trials (which we hereinafter call Event A_B or A#B). The association of contiguous events was time-locked to the presentation of the two words in the AB trials (named hereafter Event AB) (Figure 1). Besides these three major types of associative processing events, the presentation of the first word in the A_B and A#B trials, together with the calculation task, was also defined. In sum, we defined seven types of events in the estimation. Events 1-3 represented, respectively, associative processing in the AB trials (Event AB), A_B trials (Event A_B), and A#B trials (Event A#B). Events 4–6 represented, respectively, the calculation task in the AB trials (Event AB), A_B trials (Event A_B), and A#B trials (Event A#B). Event 7 represents the presentation of the first word in the A_B and A#B trials. All events were time-locked to the beginning of the stimulus presentation and modeled with a canonical hemodynamic response function (HRF) and as impulse (zero duration). The threshold for significant was set at *p* < 0.001 (uncorrected for multiple comparisons), and the threshold for cluster size was set at 100 or more continuous voxels.

## 3. Results

Mean reaction times (RTs) in each type of event are given in Table 1. There was no significant difference between the RTs of Events A#B and A_B (*t*_[11]_ = 0.96, *p* = 0.36). However, these were both significantly shorter than the RT of Event AB (Event A#B versus AB: *t*_[11]_ = 7.74, *p* < 0.01; Event A_B versus AB: *t*_[11]_ = 5.79, *p* < 0.01). The accuracy of semantic relatedness judgment is given in Table 2. In contrast with the old/new recognition task that has standard criteria, the semantic-relatedness judgment tasks were based on one's subjective “feeling” of the word pairs. Therefore, any judgment was reasonable if it was made based on the individual's careful consideration. The above-mentioned accuracy scores were just provided for reference. Subjects' posttask reports demonstrated that they had neither any difficulty nor inability to retrieve the first word in A_B and A#B trials.

**Figure 2:**
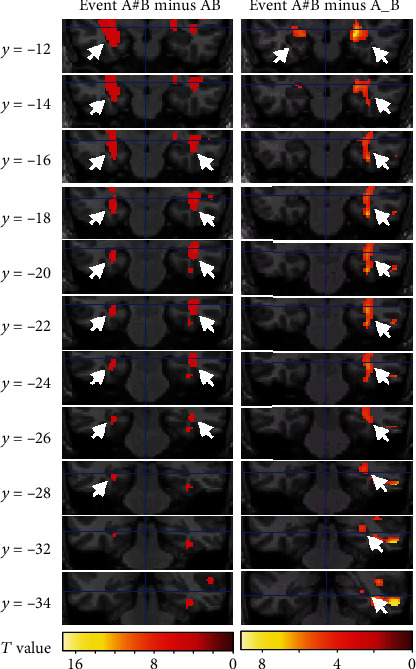
MTL activities shown when Event A#B was contrasted with Events AB and A_B, respectively. White arrows point to the hippocampal activities.

**Figure 3:**
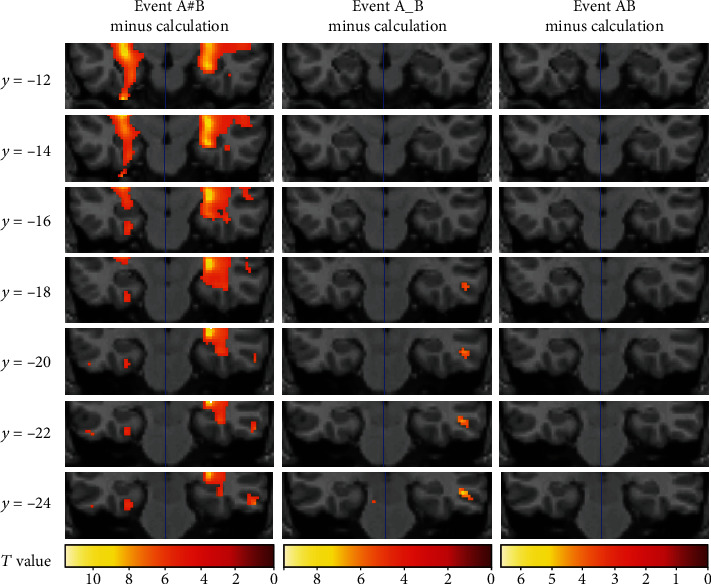
Territories of MTL activities shown when Events A#B, A_B, and AB were, respectively, contrasted with the calculation task.

We consider the effects of the delayed presentation and disturbed delayed presentation in three pairs of comparisons, i.e., “Event A#B vs. AB,” “Event A#B vs. A_B,” and “Event A_B vs. AB,” therefore consisting of a total of six contrasts. Lists of activation shown in each comparison are given in Tables 3–5. Relative to Event AB, Event A#B was associated with activations in the left side middle and superior temporal gyrus, medial and middle frontal gyrus, posterior cingulate, fusiform gyrus, lentiform nucleus, insula, amygdala, claustrum, putamen, caudate, hippocampus, and parahippocampal gyrus, as well as activations in the right side superior temporal gyrus, fusiform gyrus, middle frontal gyrus, hippocampus, and parahippocampal gyrus. Relative to Event AB, Event A_B was associated with activation in the left side paracentral lobule, postcentral gyrus, and posterior cingulate gyrus. Relative to Event A_B, Event A#B was associated with activation in left middle and superior temporal gyrus, anterior cingulate gyrus, amygdala, hippocampus, and parahippocampal gyrus and activations in right middle, superior and inferior temporal gyrus, fusiform gyrus, insula, caudate, amygdala, and hippocampus. In this experiment, we only concentrated on activation of the hippocampus and other MTL areas. Two contrasts, “Event A#B minus AB” and “Event A#B minus A_B,” revealed significant hippocampal and parahippocampal activity. These two comparisons revealed activation of the amygdala and anterior hippocampus extending into posterior hippocampal and parahippocampal regions (Figure 2). The comparison of each of the three associative processing conditions (i.e., Events AB, A_B, and A#B) and their corresponding calculation tasks yielded consistent results (Figure 3).

## 4. Discussion

In this study, we observed that the hippocampus and parahippocampus were more activated in Event A#B than in Events A_B and AB. This MTL activation could not be attributed to the semantic associative judgment itself because this process is equally involved in all three conditions. In addition, this MTL activation could not be interpreted as the bridging of temporal discontiguity because Event A_B also contained a 6 sec delay period but was not associated with more MTL activation relative to Event AB. Instead, the hippocampal activation in Event A#B implied that the hippocampus was more involved in the bridging of the “functional discontiguity” produced by the interruption task between the two to-be-combined events. Specifically, we observed hippocampal activation in two contrasts: “Event A#B minus AB” and “Event A#B minus Event A_B.” In the former contrast, besides the difference in the simultaneous and delayed presentation of the two words, Event A#B also differed from Event AB in the number of words (one or two) that participants saw in the moment of associative processing. In the latter contrast, however, the difference in the number of words no longer existed. However, Event A#B was still found to be associated more with hippocampal activation, relative to Event A_B, thus excluding that possible confounding.

This result is consistent with early studies on hippocampal amnestic patients. These patients could hold normal conversations but could not remember facts across conversations [17–19]. Or they could remember a three-figure number but would forget it the instant their attention was diverted [20]. One such particular example was the patient H.M., who could remember a number for 15 minutes by constantly rehearsing it, forgetting that he had even been given a number to remember the moment the examiner switched subjects of talk [21]. Our results are also consistent with neuroimaging studies reporting hippocampal or MTL activity in WM tasks [9, 22–25] and in delayed-match-to-sample tasks [26, 27]. For example, Elliott and Dolan [23] reported that a long delay was associated with more hippocampal activity than a short delay in a delayed-match-to-sample task and that this result could be explained as the longer delay increasing the chances of functional discontiguity occurring. In Luo and Niki's [10] experimental design that only included two conditions (AB and A_B trials), hippocampal activation was also observed in the comparison of “Event A_B minus AB.” However, in the present research that included three conditions (AB, A_B, and A#B trials), we failed to observe any significant hippocampal activation in the comparison of “Event A_B minus AB.” This implied the insertion of A#B trials across the experimental session might have altered the general processing strategy that participants took in accomplishing all the cognitive tasks including the A_B ones. They might have been more involved in the active maintenance of the first words during the delay period in the A_B condition, and this might eliminate the MTL difference in the associative processing of Event A_B relative to AB. Another difference between Luo and Niki's [10] study and the present one was in that previous study the contrast of “Event A_B minus AB” found left hippocampal activation, whereas in the present study we observed bilateral hippocampus activations in the contrasts of “Event A#B minus AB” or “Event A#B minus A_B.” The left hippocampal activation in Luo and Niki's [10] study, together with the activation of left middle temporal gyrus in that study, could be related to the more intensive processing of semantic retrieval and matching in Event A_B. In contrast, the involvement of bilateral hippocampus in Event A#B of the present study, especially the additional participation of the right hippocampus, might embody the extra processing demands on memory retrieval. In support of this possibility, the WM performance has been found to be more fragile to the damage in right lateral hippocampus relative to the left ones [28].

In the present study, we found that the bilateral hippocampus was more activated when the associative processing was carried out between the two items, which were separated by the interruptive calculation task. However, the exact cognitive components that caused this effect remain to be specified. Given that the only difference between Events A#B and A_B was the interruption of the online maintenance of the first words, we guessed it was the extra memory retrieval of the first word in Event A#B that evoked hippocampal activation. The nature of the memory retrieval involved in Event A#B, however, could be different from the ones in a typical WM task, although the delay period between the presentation of the first and second word was very brief (6 sec). First, in the typical WM retrieval, the solicited information is constantly kept online in the visual-spatial sketch pad and/or articulatory loop of the WM buffer. However, in Event A#B, the WM buffer that maintained the perceptual and semantic processing results of the first words was refreshed or updated by the interruptive calculation task [9]. Second, the memory retrieval of the first word was not triggered by a typical recognition memory or recall memory requirement but triggered by the presentation and processing of the second word. This task not only required the memory retrieval of the first word but also and even more dominantly required the extensive semantic retrieval and comparison of the first and second words.

These features inherent to our cognitive tasks made it difficult for us to further identify the neural correlates corresponding to the WM components involved in conditions A#B and A_B. This is due to the fact that both the memory retrieval of the first word and the perceptual encoding of the second word, as well as the semantic comparisons between the first and second words, cooccurred during the moment of associative processing. We have tried to identify common activation patterns involved in Events A#B and A_B by contrasting them with the AB condition but found few common activation patterns in Events A#B and A_B. This suggested that the possible common cognitive components of memory retrieval (of the first word) contained in Events A#B and A_B were rare. This could be due to the fact that individuals in the A_B condition might have tried to retrieve the first word during the delay period and at the presentation of the second word, rendering the memory retrieval component less significant. However, technically, we had no way to know the exact time at which participants started to rehearse the first word during the relay period. In addition, we did not know whether they did so in explicit or implicit ways. Therefore, it was difficult for us to further identify the neural signals that were specifically related to the process of memory retrieval during the maintenance period of the first word in conditions A#B and A_B. The only time window to check the WM components is the onset time of the second word in the A#B or A_B trials. However, at that time, the processes of semantic retrieval, selection, and comparison between the two words were also involved and mixed with the processes of memory retrieval, thus rendering us unable to ensure a reasonable disassociation.

Although computational models essentially pointed to the function of the hippocampus in discontiguity binding [2, 4], the present study found both hippocampal and parahippocampal activation. This is different from the previous study that only detected hippocampal activation [10]. A reason for the coactivation of the hippocampus and parahippocampus in functional discontiguity binding may be the extensive short-term memory and semantic memory retrieval involved in Event A#B. This conjecture was consistent with Wais et al.'s [29] study that suggested the processes of recollection and familiarity in memory retrieval that could be jointly supported by the hippocampus and parahippocampus.

While the present study used a calculation task in which the presented numbers were compared, could this comparison task leave enough room to still rehearse the first word in the A#B trials? In order to estimate whether the calculation task could prohibit the rehearsal of the first word in the A#B trials, we compared the calculation in A#B trials with that in A_B trials. Results showed that, relative to the calculation in A_B trials, the calculation in A#B trials was associated with activation in (a) left precentral gyrus (*x*, *y*, *z* = −32, −27, 47; *T* = 6.58; BA4), (b) right cuneus (*x*, *y*, *z* = 10, −68, 33; *T* = 6.04; BA7); (c) left thalamus (*x*, *y*, *z* = −8, −31, 2; *T* = 5.94), and (d) medial frontal gyrus (*x*, *y*, *z* = −2, 2, 48; *T* = 5.27; BA6). These activation patterns, especially in the medial frontal gyrus, revealed that an extra process of mental shifting was evoked in the calculation task of A_B trials. However, brain areas linked to rehearsal, including the dorsolateral prefrontal cortex, left superior temporal region, and Broca's area [9], were not detected as being activated in the comparison. This implied there was no obvious rehearsal component inherent to the calculation task in A#B trials. In addition, in the comparison of “the calculation in A#B trials minus the calculation in A_B trials,” no activation difference in the MTL region was detected. This implied that the MTL activation associated with Event A#B was transient in the moment of associative processing and did not appear during the interruption interval.

This study sought to address how to experimentally define “discontiguity” associated with hippocampal activity. The study's results showed that “discontiguity” could be more directly related to the “interruption” of the online maintenance of information held in WM than to the simple time or space “gap” (interval) between two items. Not only is this conclusion consistent with the general point of view that the function of the hippocampus is to recall a context or bridge a contextual gap, but it also provides an alternative explanation to the experimental observations that seem to be inconsistent with the discontiguity-binding hypothesis. For example, a previous study found that a discontiguous presentation of events and difficulty in performing the task inherent to the long delay interval between the conditioned stimulus and the unconditioned stimulus could impair the performance of animals with hippocampal lesions [30]. In addition, according to the implication of the present study, this phenomenon could be interpreted in light of the fact that the long interval allowed for a higher chance for functional discontiguity to occur.

This study had a few limitations. First, the sample size of the study was relatively small, although a random effect analysis was conducted and each condition contained enough trials. A larger sample size could be more reliable for drawing further conclusions. Second, as we said in the Introduction, although our study assessed the involvement of the hippocampus in discontiguity binding, no subsequent memory test was used to further examine if these bindings were successfully encoded in long-term memory or not. As such, we did not have any information on hippocampal involvement in the successful (versus unsuccessful) long-term memory storage of these discontiguity bindings. This lack of information may somewhat limit the deductive power of this study in its conclusions on the function of the hippocampus in binding the two discontiguous words and may lead to a “memory retrieval” interpretation. It implies that the memory retrieval of the first word, rather than the binding of the first and second words, may be what really contributed to the hippocampal activation in Event A#B. These components of memory retrieval, though they also occurred in the task context of associative processing, could somewhat deviate from the major concerns of the two-word binding processes. More objective measurement of the internal processing of discontiguity binding should also be considered and tested to clarify this issue.

## 5. Conclusions

In sum, in this study, we demonstrated that the hippocampus is more activated in the bridging of “functional discontiguity” than in the bridging of “temporal discontiguity.” This observation showed that the hippocampus could be mostly suited to associating items that are separated by distractors. This study lays forth additional information relevant to the theories that propose a hippocampal role in associating discontiguous items that occur in different temporal or spatial positions [2].

## Figures and Tables

**Table 1:** Mean reaction time (sec) (*N* = 12).

Event type	Event A#B	Event A_B	Event AB	Calculation (in A#B trials)	Calculation (in A_B trials)	Calculation (in AB trials)
Mean	1.47	1.44	1.64	1.56	1.61	1.61
SD	0.21	0.23	0.21	0.21	0.17	0.16

**Table 2:** Mean judgment accuracy (%) (*N* = 12).

	A#B		A_B		AB	
	Hit	CR	Hit	CR	Hit	CR
Mean	0.92	0.87	0.91	0.82	0.94	0.85
SD	0.08	0.12	0.07	0.11	0.07	0.10

Hit: the ratio of “yes” judgment to the predesigned related pairs in A#B condition; CR: the ratio of “no” judgment to the predesigned unrelated pairs.

**Table 3:** Activities shown in the contrast of “Event A#B > AB” and “Event AB > A#B”.

Voxel size	*T*	*x*	*y*	*z*	Area
*EventA*#*B* > *AB*					
4610	17.24	-26	-17	12	L. lentiform nucleus
	15.3	-28	-21	14	L. claustrum
	7.88	-32	-10	-5	L. claustrum
	6.86	-48	-65	16	L. middle temporal gyrus, BA 39
	6.84	-50	-59	16	L. superior temporal gyrus, BA 22
	6.74	-30	-15	3	L. putamen
	6.73	-18	-55	18	L. posterior cingulate, BA 30
	6.37	-30	-40	13	L. caudate
	**6.36**	**-32**	**-39**	**-3**	**L. parahippocampal gyrus, BA 19**
	6.23	-34	-51	-11	L. fusiform gyrus, BA 37
	**6.22**	**-26**	**-47**	**-6**	**L. parahippocampal gyrus, BA 19**
	6.19	-32	-42	-23	L. fusiform gyrus, BA 20
	6.18	-24	11	18	L. claustrum
	6.14	-51	-56	6	L. middle temporal gyrus, BA 39
	**6.12**	**-26**	**-46**	**10**	**L. parahippocampal gyrus, BA 30**
	6.05	-22	11	25	No GM
	6.02	-18	7	24	L. caudate
	**5.83**	**-30**	**-54**	**10**	**L. parahippocampal gyrus, BA 30**
	5.68	-32	-60	9	L. middle temporal gyrus, BA 19
	5.39	-42	-2	-5	L. insula, BA 13
	**5.26**	**-28**	**-26**	**-9**	**L. hippocampus**
	5.03	-50	-55	25	L. superior temporal gyrus, BA 39
	4.89	-14	-60	9	L. posterior cingulate, BA 30
	4.49	-40	7	-12	L. superior temporal gyrus, BA 38

291	9.17	-6	35	39	L. medial frontal gyrus, BA 8
	4.48	-10	40	26	L. medial frontal gyrus, BA 9
	4.43	-8	27	45	L. medial frontal gyrus, BA 8

211	7.79	46	-20	-7	R. superior temporal gyrus, BA 22

132	7.59	42	11	34	R. middle frontal gyrus, BA 9

218	7.29	-36	18	-23	L. superior temporal gyrus, BA 38
	5.55	-26	1	-20	L. amygdala
	5.21	-30	10	-26	L. superior temporal gyrus, BA 38
	5.06	-26	7	-24	L. superior temporal gyrus, BA 38
	4.76	-36	8	-27	L. superior temporal gyrus, BA 38
	7.29	-36	18	-23	L. superior temporal gyrus, BA 38
	5.55	-26	1	-20	L. amygdala
	5.21	-30	10	-26	L. superior temporal gyrus, BA 38

496	7.21	42	-34	-17	R. fusiform gyrus, BA 20
	**6.11**	**40**	**-26**	**-19**	**R. parahippocampal gyrus, BA 20**
	5.52	26	-46	12	No GM
	**5.00**	**32**	**-47**	**-6**	**R. parahippocampal gyrus, BA 19**

191	6.77	-6	54	-11	L. medial frontal gyrus, BA 11
	4.98	-24	41	-4	L. middle frontal gyrus, BA 11
	4.39	-26	25	2	L. claustrum

*EventAB* > *A*#*B*					
340	6.51	6	-56	-1	R. cerebellum
	4.50	4	-70	2	R. lingual gyrus, BA 18
	4.12	8	-45	-8	R. cerebellum

164	6.17	2	-1	50	R. medial frontal gyrus, BA 6

158	5.74	6	-26	-10	R. red nucleus

L.: left; R.: right; BA: Brodmann area; no GM: no gray matter is found in the 11 × 11 × 11 mm cube range. Threshold was set at *p* < 0.001 (uncorrected), KE > 100 voxels. The activities in MTL are in bold type. Note that some voxel cluster size was large and this could be related to the normalization that involved subsampling to 2 × 2 × 2 mm from the original voxel size.

**Table 4:** Activities shown in the contrast of “Event A#B > A_B” and “Event A_B > A#B”.

Voxel size	*T*	*x*	*y*	*z*	Area
*EventA*#*B* > *A*_*B*					
1207	9.15	59	-35	-10	R. middle temporal gyrus, BA 21
	6.41	26	-10	-13	R. amygdala
	**6.32**	**28**	**-12**	**-9**	**R. hippocampus**
	6.3	48	-34	-13	R. fusiform gyrus, BA 37
	5.92	48	-35	-7	R. fusiform gyrus, BA 37
	5.91	61	-43	-8	R. middle temporal gyrus, BA 21
	5.9	48	-33	3	R. superior temporal gyrus, BA 22
	5.85	38	-19	-21	R. subgyral, BA 20
	5.53	38	-22	-9	R. insula, BA 13
	4.91	61	-22	-19	R. inferior temporal gyrus, BA 20
	4.80	36	-14	-9	R. caudate
	4.78	34	-29	-4	R. caudate

520	7.11	-24	3	24	No GM
	5.41	-12	27	28	L. cingulate gyrus, BA 32
	4.28	-10	4	37	L. cingulate gyrus, BA 24

217	6.70	-48	-65	16	L. middle temporal gyrus, BA 39
	5.28	-53	-58	7	L. middle temporal gyrus, BA 39
	4.91	-40	-71	20	No GM

824	6.29	-28	-3	-18	L. amygdala
	5.60	-34	10	-24	L. superior temporal gyrus, BA 38
	5.25	-36	16	-29	L. superior temporal gyrus, BA 38
	5.22	-20	-10	-13	L. amygdala
	4.61	-44	-8	-13	L. subgyral, BA 21

336	5.14	-36	-52	6	No GM
	**4.82**	**-36**	**-47**	**-1**	**L. parahippocampal gyrus, BA 19**
	**4.76**	**-38**	**-45**	**2**	**L. parahippocampal gyrus, BA 19**
	**4.43**	**-30**	**-39**	**-3**	**L. hippocampus**
	**4.26**	**-30**	**-43**	**-10**	**L. parahippocampal gyrus, BA 37**
	4.13	-40	-37	-2	L. subgyral

*EventA*_*B* > *A*#*B*					
244	6.74	2	-1	50	R. medial frontal gyrus, BA 6

335	6.33	4	-56	1	R. cerebellum
	**4.38**	**8**	**-45**	**-6**	**R. parahippocampal gyrus, BA 30**

183	5.85	8	-26	-9	R. substantia nigra
	5.42	-4	-30	-12	No GM
	5.24	8	-28	-19	No GM

L.: left; R.: right; BA: Brodmann area; no GM: no gray matter is found in the 11 × 11 × 11 mm cube range. Threshold was set at *p* < 0.001 (uncorrected), KE > 100 voxels. The activities in MTL are in bold type.

**Table 5:** Activities shown in the contrast of “Event A_B > AB” and “Event AB > A_B”.

Voxel size	*T*	*x*	*y*	*z*	Area
*EventA*_*B* > *AB*					
321	8.34	-2	-19	45	L. paracentral lobule, BA 31
	6.03	-4	-25	40	L. cingulate gyrus, BA 31
	4.53	-2	-10	32	L. cingulate gyrus, BA 24

187	5.88	-36	-29	51	L. postcentral gyrus, BA 3
	5.31	-40	-27	44	L. postcentral gyrus, BA 40

*EventAB* > *A*_*B*					
104	5.87	-26	-52	-26	L. cerebellum
	4.76	-34	-60	-31	L. cerebellum

L.: left; R.: right; BA: Brodmann area. Threshold was set at *p* < 0.001 (uncorrected), KE > 100 voxels.

## Data Availability

The datasets are available from the corresponding author Jing Luo (luoj@psych.ac.cn).

## References

[1] Levy W. B. (1996). A Sequence predicting CA3 is a flexible associator that learns and uses context to solve hippocampal-like tasks. *Hippocampus*.

[2] Wallenstein G. V., Hasselmo M. E., Eichenbaum H. (1998). The hippocampus as an associator of discontiguous events. *Trends in Neurosciences*.

[3] Fortin N., Wright S., Eichenbaum H. (2004). Recollection-like memory retrieval in rats is dependent on the hippocampus. *Nature*.

[4] Levy W. B., Sederberg P. B. A neural network model of hippocampally mediated trace conditioning.

[5] MacDonald C. J., Lepage K. Q., Eden U. T., Eichenbaum H. (2011). Hippocampal “time cells” bridge the gap in memory for discontiguous events. *Neuron*.

[6] MacDonald C. J., Carrow S., Place R., Eichenbaum H. (2013). Distinct hippocampal time cell sequences represent odor memories in immobilized rats. *The Journal of Neuroscience*.

[7] Kitamura T., Macdonald C. J., Tonegawa S. (2015). Entorhinal–hippocampal neuronal circuits bridge temporally discontiguous events. *Learning & Memory*.

[8] Modi M. N., Dhawale A. K., Bhalla U. S. (2014). CA1 cell activity sequences emerge after reorganization of network correlation structure during associative learning. *eLife*.

[9] Sakai K., Rowe J. B., Passingham R. E. (2002). Parahippocampal reactivation signal at retrieval after interruption of rehearsal. *The Journal of Neuroscience*.

[10] Luo J., Niki K. (2005). Does hippocampus associate discontiguous events? Evidence from event-related fMRI. *Hippocampus*.

[11] Qin S., Piekema C., Petersson K. M., Han B., Luo J., Fernández G. (2007). Probing the transformation of discontinuous associations into episodic memory: an event-related fMRI study. *NeuroImage*.

[12] Liu X., Qin S., Rijpkema M., Luo J., Fernández G. (2010). Intermediate levels of hippocampal activity appear optimal for associative memory formation. *PLoS One*.

[13] Kohler S., Crane J., Milner B. (2002). Differential contributions of the parahippocampal place area and the anterior hippocampus to human memory for scenes. *Hippocampus*.

[14] Luo J., Niki K. (2002). Role of medial temporal lobe in extensive retrieval of task-related knowledge. *Hippocampus*.

[15] Henke K., Weber B., Kneifel S., Wieser H. G., Buck A. (1999). Human hippocampus associates information in memory. *Proceedings of the National Academy of Sciences*.

[16] Staresina B. P., Davachi L. (2009). Mind the gap: binding experiences across space and time in the human hippocampus. *Neuron*.

[17] Cohen N. J., Eichenbaum H. (1993). *Memory, Amnesia, and the Hippocampal System*.

[18] Milner B., Corkin S., Teuber H.-L. (1968). Further analysis of the hippocampal amnesic syndrome: 14-year follow-up study of H.M. *Neuropsychologia*.

[19] Scoville W. B. (1968). Amnesia after bilateral mesial temporal-lobe excision: introduction to case H.M. *Neuropsychologia*.

[20] Scoville W. B., Milner B. (1957). Loss of recent memory after bilateral hippocampal lesions. *Journal of Neurology, Neurosurgery, and Psychiatry*.

[21] Milner B., Pribram K. H., Broadbent D. E. (1970). Memory and the medial temporal regions of the brain. *Biology of Memory*.

[22] Curtis C. E., Zald D. H., Lee J. T., Pardo J. V. (2000). Object and spatial alternation tasks with minimal delays activate the right anterior hippocampus proper in humans. *Neuroreport*.

[23] Friedman H. R., Goldman-Rakic P. S. (1988). Activation of the hippocampus and dentate gyrus by working-memory: a 2- deoxyglucose study of behaving rhesus monkeys. *The Journal of Neuroscience*.

[24] Haxby J. V., Ungerleider L. G., Horwitz B., Rapoport S. I., Grady C. L. (1995). Hemispheric differences in neural systems for face working memory: a PET-rCBF study. *Human Brain Mapping*.

[25] Sybirska E., Davachi L., Goldman-Rakic P. S. (2000). Prominence of direct entorhinal-CA1 pathway activation in sensorimotor and cognitive tasks revealed by 2-DG functional mapping in nonhuman primate. *The Journal of Neuroscience*.

[26] Monk C. S., Zhuang J., Curtis W. J. (2002). Human hippocampal activation in the delayed matching- and nonmatching-to-sample memory tasks: an event-related functional MRI approach. *Behavioral Neuroscience*.

[27] Elliott R., Dolan R. (1999). Differential neural responses during performance of matching and nonmatching to sample tasks at two delay intervals. *The Journal of Neuroscience*.

[28] Abrahams S., Morris R. G., Polkey C. E. (1999). Hippocampal involvement in spatial and working memory: a structural MRI analysis of patients with unilateral mesial temporal lobe sclerosis. *Brain and Cognition*.

[29] Wais P. E., Wixted J. T., Hopkins R. O., Squire L. R. (2006). The hippocampus supports both the recollection and the familiarity components of recognition memory. *Neuron*.

[30] Beylin A. V., Gandhi C. C., Wood G. E., Talk A. C., Matzel L. D., Shors T. J. (2001). The role of the hippocampus in trace conditioning: temporal discontinuity or task difficulty?. *Neurobiology of Learning and Memory*.

